# In long-lasting cellular stress phases of melanoma cells, stress granules are dissolved by HSP70

**DOI:** 10.1007/s00018-025-05939-8

**Published:** 2025-10-28

**Authors:** Sebastian Staebler, Katharina Pieger, Jacqueline Perl, David Stieglitz, Aranya Thongmao, Nadja Schneider, Anja Katrin Bosserhoff, Silke Kuphal

**Affiliations:** Institute of Biochemistry, Friedrich-Alexander University Erlangen-Nürnberg, Fahrstrasse 17, 91054 Erlangen, Germany

**Keywords:** Stress granules, Melanoma, Vemurafenib-resistance, Cellular stress

## Abstract

Cancer cells must adapt to harsh microenvironmental conditions such as nutrient deprivation, hypoxia and immune pressure to sustain their high proliferative potential. One adaptive mechanism involves the formation of stress granules (SGs), which promote cell survival under stress. Interestingly, melanoma cells appear to tolerate such stressors, including hypoxia and chemotherapeutic agents, with minimal dependence on SG formation. In this study, we identify heat shock protein 70 (HSP70) as a key mediator of stress resistance in melanoma cells and the participation of the kinase CK2 in this process. We demonstrate that melanoma cells express high endogenous levels of HSP70, which preserves protein homeostasis and inhibits apoptosis under both environmental and drug-induced stress. Silencing of HSP70 led to increased SG formation and sensitized melanoma cells to apoptosis, particularly in response to the BRAF inhibitor Vemurafenib. These findings suggest that HSP70 plays a central role in melanoma cell survival by compensating for the need for SGs and promoting resistance to therapeutic stress.

**Supplementary Information:**

The online version contains supplementary material available at 10.1007/s00018-025-05939-8.

## Introduction

Melanoma is the most lethal form of skin cancer, accounting for the majority of skin cancer-related deaths [1, 2]. Among its molecular drivers, the most common initiating mutation is BRAF^V600E^, which results in constitutive activation of the BRAF kinase within the mitogen-activated protein kinase (MAPK) signaling pathway [3]. This pathway is aberrantly activated in the majority of melanoma cases and promotes uncontrolled cell proliferation [4]. For patients with BRAF-mutant stage III melanoma, current treatment guidelines recommend adjuvant therapy using BRAF inhibitors such as dabrafenib or vemurafenib in combination with the MEK inhibitor trametinib [5]. While initial responses to targeted therapies are often favorable, most patients eventually develop drug resistance [6]. One proposed mechanism underlying this resistance is the activation of cellular stress response pathways triggered by treatment [7]. In addition to classical stress responses such as autophagy, apoptosis, and DNA repair, the formation of stress granules (SGs) has emerged as a key protective mechanism [8]. Stress granules (SGs) have been observed in tumors of various entities, including colorectal cancer, pancreatic carcinoma, osteosarcoma, and glioblastoma [9–12]. SGs are dynamic, cytoplasmic, non-membranous aggregates that sequester translationally inactive messenger ribonucleoprotein particles (mRNPs) into compartments ranging from 100 to 2000 nm in size [13, 14]. SGs typically form transiently in response to stress and are disassembled once normal conditions are restored [15].

One common experimental method to induce SGs in vitro is the treatment with sodium arsenite (SA), which activates the serine/threonine kinase HRI (heme-regulated inhibitor) [13]. HRI phosphorylates eukaryotic initiation factor 2 alpha (eIF2α), leading to translational arrest and SG assembly. SG maturation requires recruitment of aggregation-prone mRNPs such as T-cell-restricted intracellular antigen-1 (TIA-1), Poly(A)-bindendes Protein (PABP), and Ras GTPase-activating protein-binding protein 1 (G3BP1) [16]. SGs can influence cancer cell behavior by modulating apoptosis, proliferation, motility, and metabolism. For example, by sequestering pro-apoptotic proteins such as RACK1, SGs may enhance cellular resistance to therapy [11, 17–20]. The involvement of SGs in therapy resistance has led to growing interest in targeting SG-related pathways to restore chemosensitivity in cancer cells [14]. However, the precise role of SGs in melanoma progression and treatment resistance remains poorly understood.

In this study, we report that melanoma cells exhibit a minimal dependence on SG formation either by limited capacity to form SGs or by a rapid disassembly following SG formation. We observed high steady-state levels of serine 149-phosphorylated G3BP1 (P-G3BP1) in melanoma cell lines, in contrast to lower levels in pancreatic (Panc-1), hepatic (Hep3B, HepG2), and colorectal (HT29) carcinoma cells. Additionally, melanoma cells showed elevated endogenous levels of heat shock protein 70 (HSP70). Both p-G3BP1 and HSP70 may contribute to impaired SG formation or enhanced SG disassembly. Notably, silencing of HSP70 in vemurafenib-resistant SK-Mel-28 R cells induced SG formation and re-sensitized the cells to treatment, suggesting a previously unrecognized role for HSP70 in modulating therapy resistance through SG regulation.

## Materials and methods

### Cell lines and cell culture conditions

Normal Human Epidermal Melanocytes derived from neonatal foreskin (NHEM, PromoCell, Heidelberg, Germany) were cultivated in M2 melanocyte growth medium (M2 with Supplementary Mix containing growth factors and hormones; PromoCell, Heidelberg, Germany) under a humidified atmosphere of 5% CO_2_ at 37 °C. They were used between Passages 4 and 14 and split once per week at a ratio of 1:4. The primary human melanoma cell line Mel Juso was cultivated in the Roswell Park Memorial Institute culture medium (RPMI) supplemented with 10% fetal bovine serum (FBS), penicillin (400 U/mL), streptomycin (50 µg/mL) and 0.2% sodium bicarbonate (all from Sigma-Aldrich, Steinheim, Germany). Metastatic melanoma cell line Mel Im was incubated at 37 °C in a 5% CO_2_ humified atmosphere low-glucose Dulbecco’s Modified Eagle’s Medium (DMEM) supplemented with 10% FBS, penicillin (400 U/mL) and streptomycin (50 µg/mL) (Sigma-Aldrich, München, Germany). All human cell lines have been authenticated using Short Tandem Repeat (STR) profiling within the last three years (DSMZ, Braunschweig, Germany and Multiplexion, Heidelberg, Germany). All cell lines are mentioned with their cell culture conditions in the Supplementary Data. All experiments were performed with mycoplasma-free cells (MycoSEQ mycoplasma detection system, Thermo Fisher Scientific, Waltham, Massachusetts, USA). Vemurafenib sensitive (SK-MEL-28NR, 451LuNR) and vemurafenib resistant (SK-MEL-28VR, 451LuVR) cell clones were a kind gift from Meenhard Herlyn, The Wistar Institute, Philadelphia, USA [21]. During culture, the resistant cell clones were permanently incubated with 0.5 µM vemurafenib (Active Biochem LTD, Bonn, Germany) to sustain drug resistance. The human colorectal adeno-carcinoma cell lines SW480 and HT29, the human pancreatic cancer cell line Panc-1 and the human hepatocellular carcinoma cell lines Hep3B and HepG2 were obtained from and cultured as indicated by the American Type and Culture Collection (ATCC).

## Induction of hypoxia and cellular stress

For the experiments under hypoxic conditions, the cells were cultivated in a water vapor-saturated atmosphere in an incubator under 1% O_2_, 5% CO_2_ and equilibrating N_2_ at 37° C. The hypoxia of 1% vol/vol, corresponding to a PO_2_ of 7 mmHg, was controlled and kept stable by a regularly calibrated oxygen sensor which is a special device in the incubator. Chemical induction of hypoxia was performed by incubating the cells with 100 µM 2–2’-Dipyridyl (DP), 100 µM Desferrioxamine (DFX) and 500 µM Dimethyloxalylglycine (DMOG), respectively, in the growth medium for 24 h prior to the experiments. For induction of cellular stress, the cells were also incubated with 2-(N-morpholino)ethanesulfonic acid (MES) and hydrogen peroxide (H_2_O_2_), respectively for 24 h. Arsenite treatment was performed using 600 µM sodium arsenite (SA) for the respective time period.

## Inhibition of CK2

Melanoma cells were grown in culture medium and pretreated for 2 h with 60 µM TBCA (Tetrabromocinnamic Acid; Cayman Chemicals) prior to arsenite stress experiments. Then, c ells were stressed for 45 min with 50 µM sodium arsenite in medium. After stress induction, cells were washed once with PBS, and the medium was replaced with fresh growth medium equilibrated to 37 °C for 24 h.

## UV-treatment of melanocytes

UVB radiation of Normal Human Epidermal Melanocytes (NHEM) (PromoCell, Heidelberg, Germany) was performed with defined UVB doses (80 mJ/cm^2^) in a UVB Transluminator (FLX-20.M Transluminator, Biometra GmbH, Göttingen, Germany). Therefore, cells were seeded out in six-well plates. On the following day, cells were treated with 600 µM sodium arsenite or control for 30 min and subsequently treated with UVB radiation. Afterwards, the irradiated cells were fixed in 4% PFA/1x PBS and further processed for immunofluorescence stainings.

## SiRNA transfection

Cells were transfected with siRNAs against HSP70 by using the Lipofectamine RNAiMAX reagent (Life Technologies, Darmstadt, Germany). 100 pmol siRNA was diluted into RNAiMAX and the siRNA was incubated for 24 h before the experiments. The siRNA oligos used in this work are listed below and are designed after the protocol by [18]: sictrl:5`-UUCUCCGAACGUGUCACGUTT-3`; siHSP70-1#:5`CCAAGCAGACGCAGAUCUUTT-3`; siHSP70-2#: 5`-CGGUUUCUACAUGCAGAGATT-3`.

### Human tissue samples

All paraffin fixated tissue samples of human melanomas were provided from the tissue bank of the Institute of Pathology, University of Regensburg, Germany. Sampling and handling of patient material were carried out in accordance with the ethical principles of the Declaration of Helsinki. The use of human tissue material had been approved by the local ethics committee of the University of Regensburg (application number 09/11 and 03/151). A tissue micro array containing 30 samples from either melanocyte derived benign nevi, or melanoma tumors was used for immunofluorescence analysis.

## Immunofluorescence analysis of cells

Immunofluorescence stainings were performed as described elsewhere [13]. Briefly, 20.000 cells were seeded in chamber slides and fixed with 4% paraformaldehyde (PFA) in 1x PBS for 15 min at room temperature. Afterwards, cells were permeabilized using ice cold (−20 °C) methanol for 10 min at room temperature and blocked for 1 h using 1% bovine serum albumin (BSA) in 1x PBS. Thereafter, 40 µl of the respective primary antibody (Supplementary Table 1) were added and cells were incubated at 4 °C over night. After washing with 1x PBS, the cells were incubated with a secondary antibody (Supplementary Table 1) for 2 h at room temperature. Finally, cells were incubated in DAPI solution (Merck KGaA, Darmstadt, Germany) in 1% BSA/1x PBS for 30 min and subsequently mounted with Aqua-Poly/Mount (US Headquarters Polysciences, Warrington, USA). Immunofluorescence staining was analyzed with an IX83 microscope (Olympus, Hamburg, Germany).

## Immunofluorescence analysis of paraffin embedded tissue sections

Paraffin of 5 μm tissue sections was removed after standard protocols (xylol 3 × 10 min, 100% isopropanol 2 × 3 min, 96% ethanol 2 × 3 min and 70% ethanol 3 min). Antigen retrieval was carried out with 0,01 M sodium citrate buffer (pH 7.2) for 40 min at 90 °C before sections were permeabilized with ice cold methanol (−20 °C) for 10 min at room temperature and subsequently blocked with 20% BSA/PBS for 20 min. Primary antibody was added and incubated at 4 °C overnight. Tissue sections were rinsed with PBS and incubated with the respective secondary antibody (Table 1) for 1 h at 37 °C, followed by rinsing with PBS and mounting with Vectashield Hard SetMounting Medium with DAPI H-1500 (Vector Laboratories, Burlingame, CA, USA).

### Western blot analysis

Western blot analysis were performed as previously described [22]. Briefly, 20 to 40 µg of total RIPA lysates were loaded onto polyacrylamide gels and subsequently blotted onto a PVDF membrane. After blocking for 1 h with 5% (skimmed milk powder) SKM/TBS-T at room temperature, membranes were incubated with primary antibodies (diluted in 3% SKM/TBS-T) (Supplementary Table 1) overnight at 4 °C. The immunoreactions were visualized by ECL staining (Bio-Rad, Feldkirchen, Germany). The densitometry was performed using the LabImage programme. Densitometric analysis is depicted without statistical evaluation, since individual measurements from individual PVDF membranes can deviate strongly from each other.

### Flow cytometry

Apoptosis was detected by Annexin V-FITC and propidium iodide (PI) staining using flow cytometry. After treatment and incubation, cells were detached as described above. Both cell culture medium and PBS used for washing were collected and combined with the cell suspension. Cells were then washed once with PBS and immediately stained using Annexin V-FITC Apoptosis Kit (Invitrogen) according to manufacturer’s instructions. Samples were analyzed using a BD LSRFortessa™ flow cytometer in combination with BD FACSDiva™ software (Version 8.0, BD Biosciences, San Jose, CA, USA).

### Quantification of stress granules

Stress granules were quantified using the cellSens software provided by Olympus. Images were acquired by randomly choosing 3 different fields of view at 20 times magnification with at least 10 cells per field of view. Cells were defined as regions of interest (ROI) using the distribution of EIF2S1 throughout the cell (minimum intensity: 10; minimum ROI area: 100 μm. The plug-in ‘‘Count and Measure’’ was used to quantify the stress granule area per total cell area in SG positive cells in each field of view. The so-called SG index was determined by computing the total stress granule area in relation to the total cell area for all fields. Additionally, cells without SGs were counted and analyzed in relation to the total number of cells in each experiment.

### Bioinformatics

For analysis of CSNK2a1 (CK2α) and G3BP1 expression GEPIA database (http://gepia.cancer-pku.cn/index.html) was used, retrieved on August 25, 2025.

### Statistical analysis

Statistical analysis was performed using GraphPad Prism software Version 10 (GraphPad Software, Inc., San Diego, CA). This software was also used to create the graphs. The results are calculated as the mean ± SEM (range) or percent. If not otherwise stated, at least 3 biological replicates were measured. Comparison between two groups was determined using unpaired Student’s t-test or One-way-ANOVA. A p-value < 0.05 (*: *p* < 0.05) was considered statistically significant (n.s.: not significant; *: *p* < 0.05; **: *p* < 0.005; ***: *p* < 0.001; ****: *p* < 0.0001).

### Data Availability

The RNA-sequencing data used in this study have been deposited in the NCBI BioProject database (https://www.ncbi.nlm.nih.gov/bioproject/) and can be accessed with the BioProject accession number PRJNA839865.

## Results

### G3BP1 expression and stress granule formation in melanoma cells

**Fig. 1:**
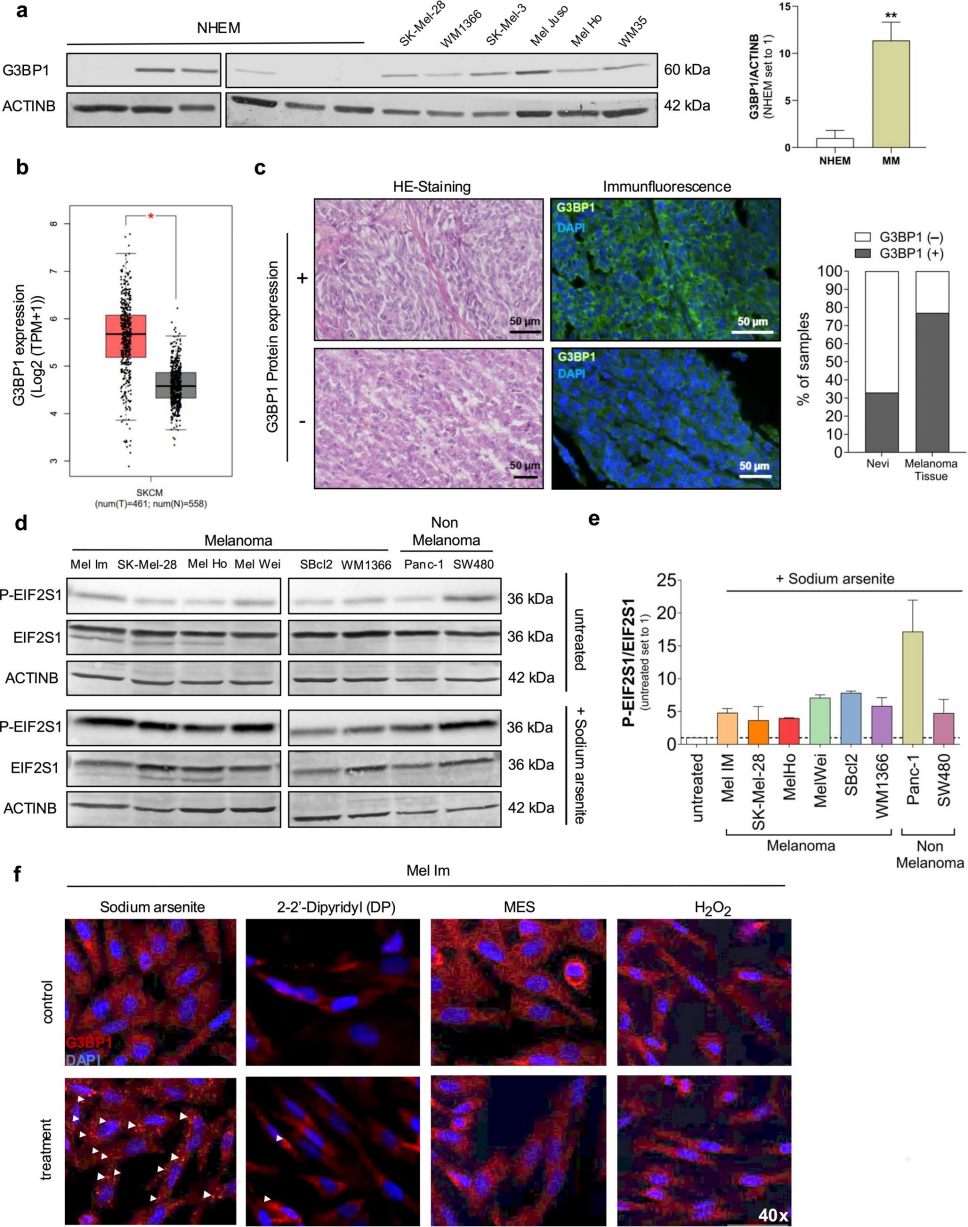
G3BP1 and (phospho-)EIF2S1 expression in malignant melanoma. (**a**) Western blot analysis of G3BP1 protein expression in normal human epidermal melanocytes (NHEM) from different donors and passages (P5–P14), and in melanoma cell lines derived from metastases (SK-Mel-28, SK-Mel-3) and primary tumors (WM1366, Mel Juso, Mel Ho, WM35). ACTINB served as a loading control. Densitometric quantification of G3BP1 level is shown. (**b**) Analysis of G3BP1 expression in skin cutaneous melanoma (SKCM) samples (red) compared to non-tumor samples (grey). Data obtained from GEPIA database, p-value cut-off = 0.01, |Log2FC| Cut-off = 1, retrieved on August 21, 2025. **(c)** Immunofluorescence staining of G3BP1 (green) in tissue sections of malignant melanoma, illustrating representative samples with positive (+) and negative (–) G3BP1 staining. DAPI (blue) was used for nuclear counterstaining. Hematoxylin and eosin (HE) staining was performed to visualize tissue morphology. Quantification of G3BP1-positive and -negative samples in tissue microarrays (nevi: *n* = 12; melanoma: *n* = 13) is shown. (**d**) Western blot analysis of phosphorylated (P-) and total EIF2S1 in melanoma and non-melanoma cell lines following sodium arsenite treatment (+ SA) (600 µM) or under untreated conditions. **(e**) Quantification of the P-EIF2S1/EIF2S1 ratio in each cell line (mean of *n* = 3 biological replicates). (**f**) Immunofluorescence staining of G3BP1 in Mel Im melanoma cells following treatment with 600 µM sodium arsenite (SA), 2,2′-Dipyridyl (DP), 2-(N-morpholino)ethanesulfonic acid (MES), or hydrogen peroxide (H₂O₂), to assess stress-induced SG formation. Magnification: 40x. Data are presented as mean ± SEM (**p < 0.01)

G3BP1 is a well-established marker for stress granule (SG) formation because of its aggregation into phase-dense cytoplasmic structures during stress [13]. Western blot analysis showed high endogenous G3BP1 expression across all tested melanoma cell lines in whole protein lysates, while normal human epidermal melanocytes (NHEM) displayed variable levels between six different donors (Fig. 1a). To further validate this observation, we analyzed data from the GEPIA dataset, which revealed significantly elevated G3BP1 expression in skin cutaneous melanoma (SKCM) samples compared to non-tumor tissues (Fig. 1b). Immunofluorescence staining revealed G3BP1 positivity in ~ 75% of melanoma tissues, compared to ~ 30% of nevi (Fig. 1c). To test the stress sensibility in melanoma, cells were treated with sodium arsenite (SA), a known stress and SG inducer. SA treatment led to increased phosphorylation of EIF2S1, indicating activation of stress response (Fig. 1d, e). Stress response to SA was also detectable in non-melanoma cell lines Panc-1 and SW480 (Fig. 1d).

Furthermore, in Mel Im cells, SG formation, marked by punctate G3BP1 staining, was strongly induced by SA, moderately by 2,2′-Dipyridyl (DP; iron chelator inducing hypoxia), and not observed with 2-(N-morpholino) ethanesulfonic acid (MES; causing a pH change) or H₂O₂ (induces oxidative stress) treatment (Fig. 1f). Similar patterns were confirmed in melanoma cell lines SBcl2 and 451Lu, as well as non-melanoma cell lines SW480 and Panc-1 (Supplementary Fig. 1). While G3BP1 was consistently expressed, SG formation was only evident under strong oxidative stress, explicit by sodium arsenite, indicating that SG assembly in melanoma is a tightly regulated response to extreme stress conditions.

### Hypoxic conditions do not robustly induce stress granule formation in melanoma cells

**Fig. 2:**
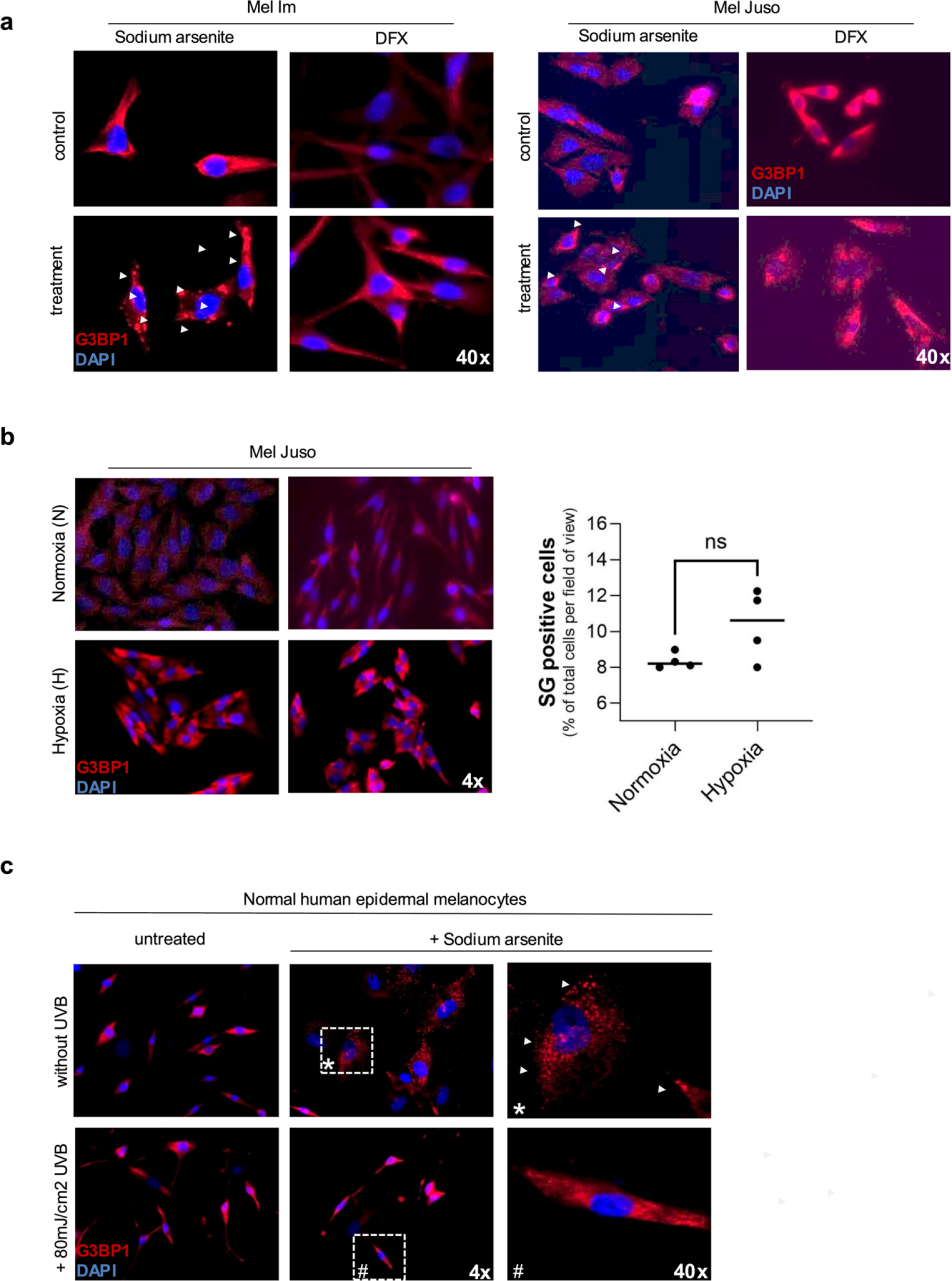
Stress granule (SG) formation in response to hypoxic, oxidative, and osmotic stress.** (a)** Immunofluorescence staining of G3BP1 in melanoma cell lines Mel Juso and Mel Im following treatment with 600 µM sodium arsenite (SA) or Desferrioxamine (DFX), an iron chelator that mimics hypoxia. SG formation is indicated by punctate G3BP1-positive structures. **(b)** G3BP1 immunofluorescence staining of Mel Juso cells incubated under hypoxic (1% O₂) or normoxic (21% O₂) conditions for 24 h. SG formation was quantified as the percentage of G3BP1-positive cells. Data represent the mean ± SEM from three independent experiments (unpaired Student’s t-test; ns = not significant). **(c)** Immunofluorescence staining of G3BP1 in normal human epidermal melanocytes (NHEM) treated with or without 600 µM sodium arsenite, under control conditions or following exposure to 80 mJ/cm² UVC irradiation (image sections *, #: 40x magnification)

Given the modest induction of stress granules (SGs) observed following DP treatment, we next examined hypoxia as a potential SG-inducing stressor. Surprisingly, chemical simulation of hypoxia using the iron chelator desferrioxamine (DFX), which promotes HIF-1α stabilization, or the prolyl hydroxylase inhibitor dimethyloxalylglycine (DMOG), failed to induce SG formation in either of the melanoma cell lines examined (Fig. 2a). Western blot analysis confirmed the stabilization of HIF-1α, validating the induction of hypoxia-like conditions (Supplementary Fig. 2a). To exclude potential artifacts associated with chemical treatments, Mel Juso and Mel Im cells were subjected to physiological hypoxia (1% O₂). The upregulation of ANGPTL4 mRNA expression served as an internal control to confirm the hypoxic response (Supplementary Fig. 2b) [23]. Consistent with the results of chemical treatment, true hypoxic conditions did not significantly induce SG formation in melanoma cells (Fig. 2b). Interestingly, sodium arsenite treatment robustly induced SGs in healthy normal human epidermal melanocytes (NHEMs) (Fig. 2c). Interestingly, while sodium arsenite alone promoted SG formation, post-SA-treatment-irradiation of NHEMs with ultraviolet B (UVB) radiation appeared to eliminate this SG response, suggesting a stress-protective effect of UVB in melanocytes, which could be conditioned by intrinsic reactions. Collectively, these results indicate that neither chemically induced (DP, DFX, DMOG) nor physiologically induced (1% O₂) hypoxia is sufficient to promote significant SG formation in melanoma cells. Furthermore, additional stressors such as pH alteration (MES buffer) and oxidative stress (H₂O₂) also failed to elicit a notable SG response, underscoring the relative insensitivity of melanoma cells to SG induction under these conditions.

### HSP70 prevents SG formation in melanoma cells

**Fig. 3:**
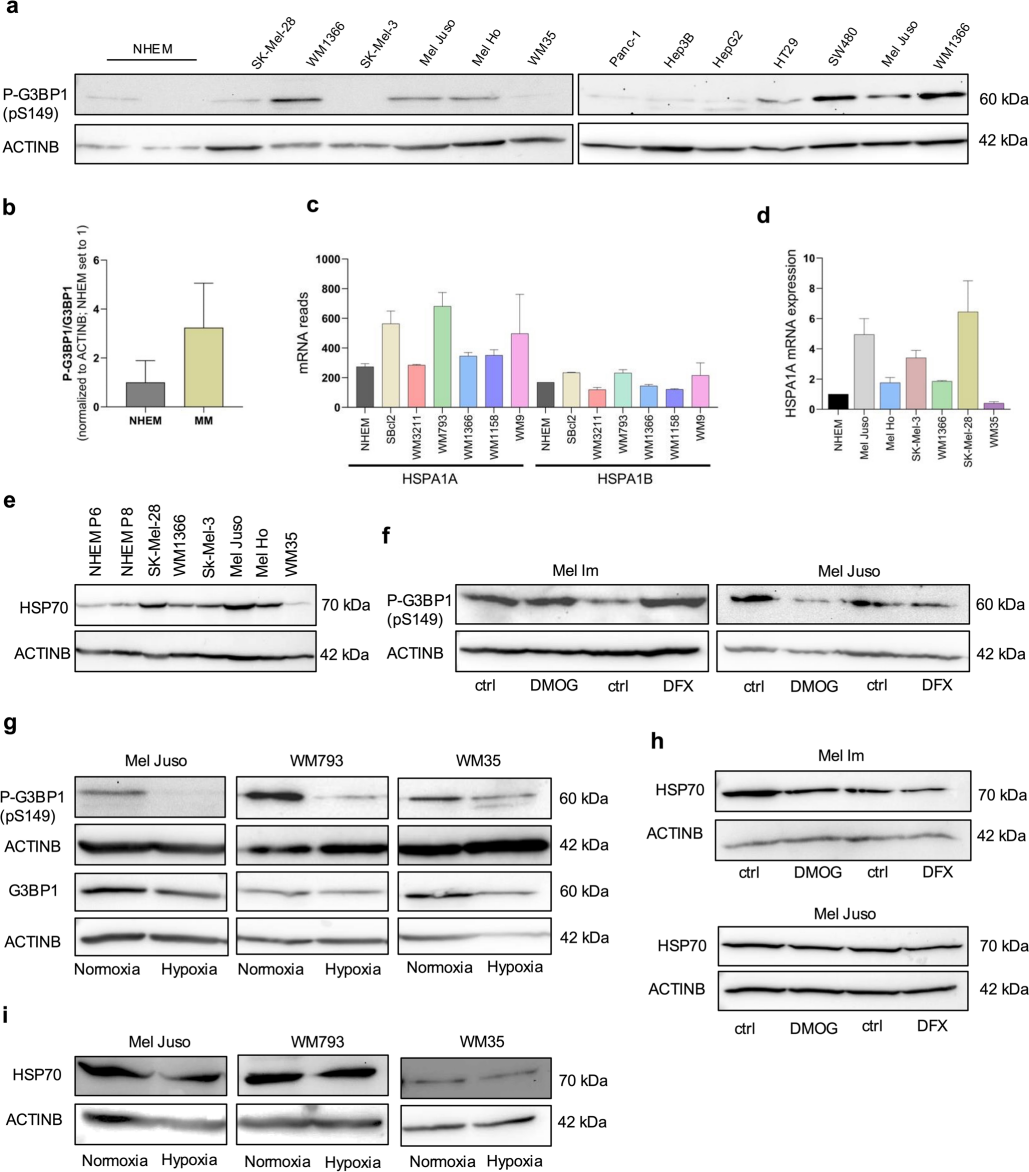
Melanoma cells exhibit high levels of HSP70 and p-G3BP1.**(a)** Western blot analysis of phosphorylated G3BP1 (P-G3BP1, Ser149) in normal human epidermal melanocytes (NHEM; passages P5 and P6) and melanoma cell lines. Additional tumor cell lines including Panc-1 (pancreatic carcinoma), Hep3B and HepG2 (hepatocellular carcinoma), and HT29 and SW480 (colorectal carcinoma) were included for comparison. ACTINB served as a loading control. **(b)** Densitometric quantification of P-G3BP1/G3BP1 protein ratio in NHEM, SK-Mel-28, WM1366, Mel Ho, Mel Juso, Sk-Mel-3 and WM35 cells. NHEM protein expression was set to 1. Normalized to ACTINB. **(c)** RNA sequencing analysis of HSP70 paralogues (HSPA1A, HSPA1B) in melanoma cell lines and NHEM. Read counts were normalized to library size. **(d)** Quantitative real-time PCR of HSPA1A and HSPA1B mRNA levels in melanoma cell lines and NHEM. **(e)** Western blot analysis of total HSP70 protein expression in NHEM (passages P5 and P8) and melanoma cell lines. ACTINB served as a loading control. **(f)** Western blot analysis of p-G3BP1 expression following treatment of Mel Juso and Mel Im cells with hypoxia-mimetic agents Desferrioxamine (DFX) and Dimethyloxalylglycine (DMOG). ACTINB was used as a loading control. **(g)** Western blot analysis of P-G3BP1 and G3BP1 expression in Mel Juso cells exposed to hypoxic conditions (1% O₂). ACTINB served as loading controls. **(h)** Western blot analysis of HSP70 expression in Mel Juso and Mel Im cells treated with DFX and DMOG. ACTINB served as a loading control. **(i)** Western blot analysis of HSP70 expression in Mel Juso cells under hypoxic conditions (1% O₂). ACTINB was used as a loading control

We now hypothesized that melanoma cells may actively prevent or rapidly resolve SGs to evade apoptosis in response to protein misfolding or other cellular stressors. Phosphorylation of G3BP1 at serine 149 (pS149) has been reported to inhibit SG assembly [24, 25]. Supporting this mechanism, we observed markedly elevated levels of phosphorylated G3BP1 (P-G3BP1) in melanoma cell lines compared to hepatocellular carcinoma (HepG2, Hep3B) and pancreatic carcinoma (Panc-1) cell lines. In contrast, colon carcinoma cell lines (HT29, SW480) exhibited a heterogeneous P-G3BP1 expression pattern (Fig. 3a). Ratio of phosphorylated G3BP1 (P-G3BP1) and G3BP1 protein level were elevated in melanoma cell lines (Fig. 3b). Additionally, literature evidence suggests that the heat shock genes Hspa1a/Hspa1b, encoding two HSP70 paralogs, play a role in SG dynamics, including their formation and clearance [18]. RNA sequencing data revealed high transcript levels of Hspa1a/Hspa1b, a duplicated HSP70 locus located in the class III region of the major histocompatibility complex on chromosome 6p21.3 [26], across several melanoma cell lines (Fig. 3c). These findings were validated by qRT-PCR, which confirmed robust expression of HSPA1A mRNA (Fig. 3d) and by Western blotting, which showed elevated HSP70 protein levels in most melanoma cell lines compared to normal human epidermal melanocytes (NHEMs) (Fig. 3e). To determine whether hypoxia influences the expression of P-G3BP1 or HSP70, we subjected melanoma cells to both chemically induced hypoxia (DFX, DMOG) and real hypoxic conditions (1% O₂). Neither treatment significantly altered P-G3BP1 expression (Fig. 3f and g), nor did they affect HSP70 protein levels in Mel Juso and Mel Im cells (Fig. 3h). This pattern was consistent under both normoxic and hypoxic conditions in multiple melanoma cell lines (Mel Juso, WM793, WM35) (Fig. 3i). Together, these results suggest that high basal levels of P-G3BP1 and HSP70 in melanoma cells may underlie their resistance to SG formation, potentially contributing to cellular stress tolerance and survival under adverse conditions.

### HSP70 suppression enhances stress granule formation and apoptotic signaling in melanoma cells

**Fig. 4:**
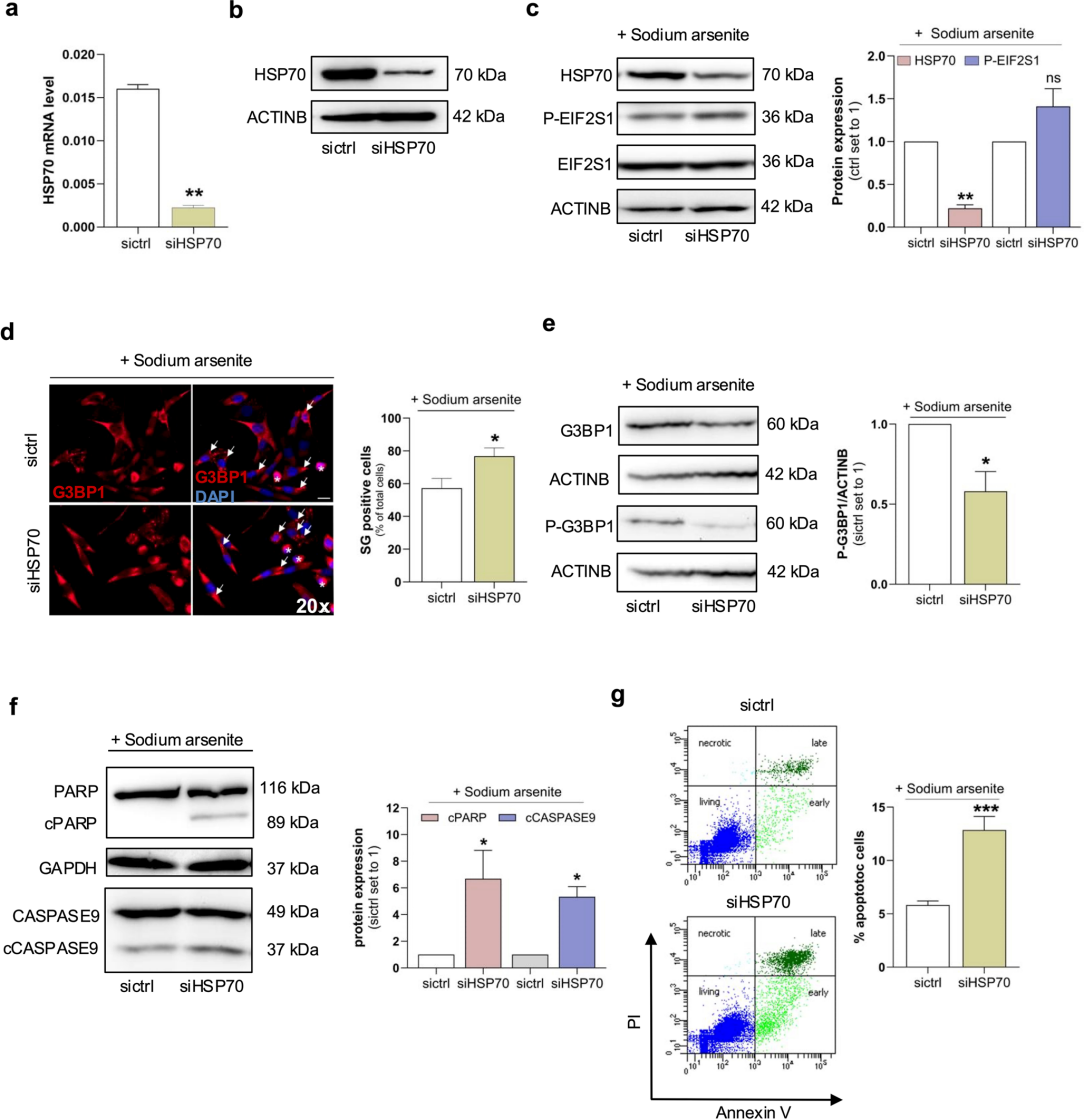
HSP70 suppresses stress granule (SG) formation and protects melanoma cells from stress-induced apoptosis. **(a)** Quantitative real-time PCR analysis of HSP70 (HSPA1A/B) mRNA levels in Mel Juso cells 24 h after transfection with an HSP70 siRNA pool (siHSP70). **(b)** Western blot analysis of HSP70 protein levels in Mel Juso cells following siHSP70 transfection for 24 h. ACTINB served as a loading control. **(c)** Western blot analysis of HSP70, phosphorylated EIF2S1 (P-EIF2S1), and total EIF2S1 in Mel Juso cells transfected with siHSP70 for 24 h and subsequently treated with 600 µM sodium arsenite (SA) to induce stress. EIF2S1 served as the internal control. **(d)** Immunofluorescence staining of G3BP1 (red) in Mel Juso cells transfected with either siHSP70 or non-targeting control siRNA (sictrl), followed by SA treatment. Arrows indicate SG formation. Quantification of G3BP1-positive cells is shown; data represent the mean of three independent experiments. **(e)** Western blot analysis of total G3BP1 and phosphorylated G3BP1 (P-G3BP1) in Mel Juso cells after transfection with siHSP70 or sictrl and SA treatment. ACTINB was used as a loading control. Densitometric quantification from three independent experiments is shown. **(f)** Western blot analysis of PARP/cleaved PARP and CASPASE9/cleaved CASPASE9 in Mel Juso cells transfected with siHSP70 or sictrl, followed by SA treatment. Full-length PARP and CASPASE9 served as loading controls. Densitometric analysis from three independent experiments is provided. **(g)** Flow cytometric analysis of apoptotic Mel Juso cells after transfection with siHSP70 or sictrl and subsequent SA treatment. Data are presented as mean ± SEM (*p < 0.05, **p < 0.01 and ***p < 0.001)

To further elucidate the role of HSP70 in stress granule (SG) regulation, we performed siRNA-mediated knockdown of HSP70 in melanoma cells (Fig. 4a and b). Notably, HSP70 silencing resulted in enhanced phosphorylation of EIF2S1 following sodium arsenite (SA) treatment, indicating increased translational stress (Fig. 4c). Immunofluorescence analysis of SA-treated Mel Juso cells revealed a marked increase in SG formation upon HSP70 knockdown (siHSP70) compared to control siRNA-treated cells (sictrl) (Fig. 4d). Consistent with this observation, Western blot analysis showed a concurrent decrease in total G3BP1 and its phosphorylated form (P-G3BP1), further implicating HSP70 in SG regulation (Fig. 4e). To investigate the link between HSP70 suppression, SG dynamics, and apoptotic signaling, we conducted Western blot and flow cytometry analyses. Combined HSP70 silencing and SA treatment resulted in increased expression of cleaved PARP and cleaved caspase-9 (cPARP, cCASP9), indicating activation of apoptosis (Fig. 4f). This was further supported by FACS analysis, which demonstrated enhanced apoptotic cell death in SA-treated siHSP70 cells compared to controls (Fig. 4g). Together, these findings suggest that HSP70 plays a protective role in melanoma cells by modulating SG formation and preventing apoptosis under stress conditions.

### Caseinkinase 2a regulates stress granule formation by modulating G3BP1 phosphorylation

**Fig. 5:**
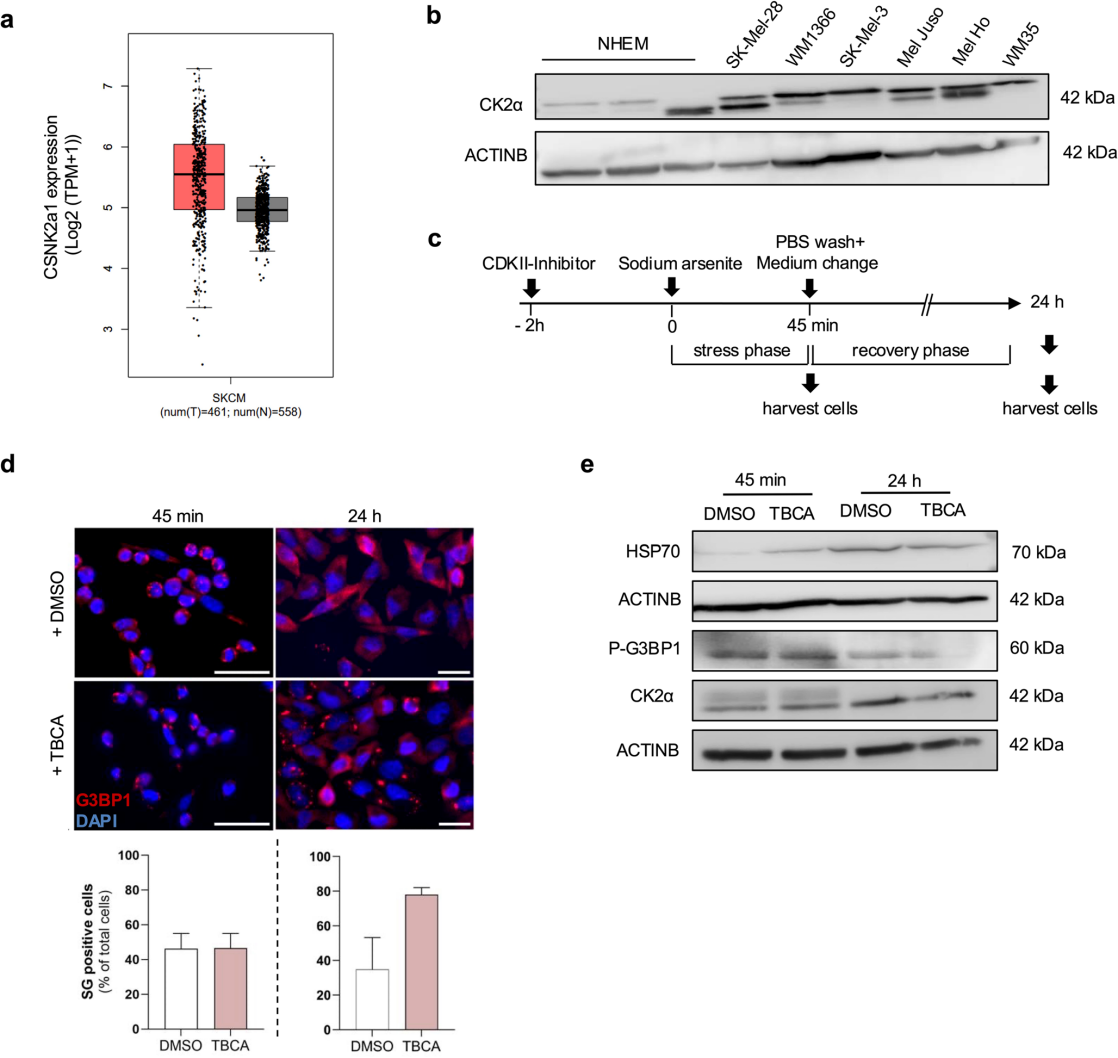
Caseinkinase 2a regulates Stress Granule Formation by modulating G3BP1 phosphorylation** (a)** Analysis of CSNK2A1 expression in skin cutaneous melanoma (SKCM; T) samples (red) compared to non-tumor skin (N) samples (grey). Data were obtained from the GEPIA database with a p-value cut-off of 0.01 and |Log₂FC| cut-off of 1 (retrieved August 21, 2025). **(b)** Representative western blot analysis of casein kinase 2α (CK2α) expression in normal human epidermal melanocytes (NHEMs) from different donors and melanoma cell lines (SK-MEL-28, WM1366, SK-MEL-3, Mel Juso, Mel Ho, and WM35). ACTINB was used as a loading control. **(c)** Schematic representation of the experimental timeline for TBCA treatment. **(d)** Immunofluorescence staining of G3BP1 (red) in Mel Juso cells treated with TBCA or DMSO for 45 min (stress phase) or after 24 h (recovery phase). Nuclei were counterstained with DAPI (blue). Quantification of stress granule (SG)-positive cells per field of view is shown as the mean of three independent experiments (lower panels). **(e)** Representative western blot analysis of HSP70, phosphorylated G3BP1 (P-G3BP1), and CK2α in cells treated with TBCA or DMSO, followed by sodium arsenite (SA) treatment for 45 min (stress phase) or 24 h (recovery phase). ACTINB served as a loading control

Since phosphorylation of G3BP1 is a critical step in suppressing stress granule (SG) formation, we next sought to identify the kinase responsible for this regulation. An in-depth literature review highlighted a study by Reineke et al. (2017), which identified Casein Kinase 2α (CK2α) as an upstream regulator of G3BP1 phosphorylation, thereby inhibiting SG assembly in osteosarcoma cells [25]. Notably, CK2α has also been reported to promote vemurafenib resistance in a kinase-independent manner in melanoma cells [27]. Analysis of publicly available datasets (GEPIA) revealed that CSNK2A1 expression is elevated in melanoma tumors compared to non-tumor tissues (Fig. 5a). Consistently, Western blot analysis confirmed high CK2α protein expression in melanoma cell lines (Fig. 5b). To examine the role of CK2α in SG regulation in melanoma cells, we treated cells with the selective CK2α inhibitor tetrabromocinnamic acid (TBCA) prior to sodium arsenite (SA)-induced stress, followed by a recovery phase (Fig. 5c). After the acute stress phase, no significant differences in SG formation were observed between control and CK2α-inhibited cells **(**Fig. 5d). However, during the recovery phase, CK2α inhibition resulted in enhanced SG formation, whereas cells with active CK2α displayed fewer SGs (Fig. 5d). In line with these observations, inhibition of CK2α reduced HSP70 and phosphorylated G3BP1 levels without altering total CK2α expression (Fig. 5e).

#### Vemurafenib-Resistant melanoma cells exhibit reduced stress granule formation under combined stress conditions

**Fig. 6:**
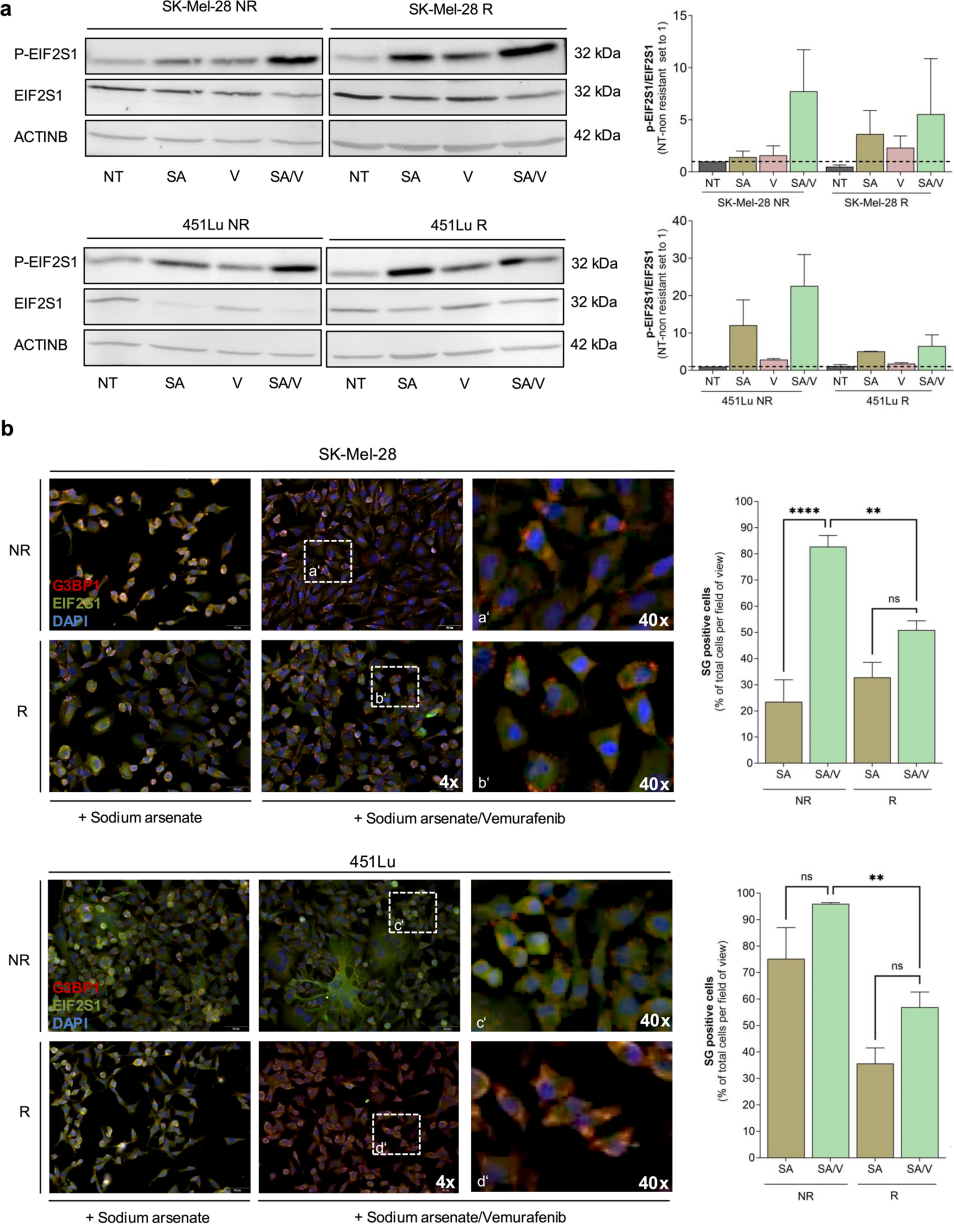
Phosphorylated EIF2S1 and stress granule (SG) dynamics in BRAF-mutant melanoma cell lines.** (a)** Representative western blot analysis of phosphorylated (p-) and total EIF2S1 in BRAF-mutant melanoma cell lines SK-Mel-28 and 451Lu, comparing non-resistant (NR) and vemurafenib-resistant (R) cells. Treatments included no treatment (NT), sodium arsenite (SA), vemurafenib (V), or a combination of both (SA/V). ACTINB was used as a loading control. Bar graph depicts the ratio of p-EIF2S1 to total EIF2S1 based on densitometry from three independent experiments. **(b)** Immunofluorescence staining of G3BP1 (red) and EIF2S1 (green) in SK-Mel-28 and 451Lu NR and R cells after treatment with SA or SA/V. Nuclei were counterstained with DAPI (blue). Quantification of SG-positive cells per field of view is shown as the mean of three independent experiments. Data are presented as mean ± SEM (*p < 0.05, **p < 0.01, ***p < 0.001 and ****p < 0.0001; ns = not significant)

We hypothesized that resistance to selective RAS-pathway inhibitors such as Vemurafenib (V) may be associated with altered stress granule (SG) dynamics. Therefore, we analyzed SG responses in Vemurafenib-resistant melanoma cell lines (451LuR and SK-Mel-28R) and their respective parental (non-resistant) controls. Cells were treated with sodium arsenite (SA), Vemurafenib (V), or a combination of both (SA/V). Western blot analysis revealed that non-resistant cells exhibited a higher P-EIF2S1 to total EIF2S1 ratio following combined SA/V treatment, indicative of elevated cellular stress. In contrast, resistant cell lines showed a blunted phosphorylation response under the same conditions. Vemurafenib treatment alone had minimal effect on P-EIF2S1 levels across all lines (Fig. 6a). Immunofluorescence staining for G3BP1 and EIF2S1 further supported these findings. Both resistant and non-resistant cell lines showed increased SG formation in response to SA/V compared to SA alone. However, Vemurafenib-resistant cells consistently exhibited a significantly lower percentage of SG-positive cells under combined stress treatment (Fig. 6b). Together, these data suggest that Vemurafenib resistance in melanoma cells is associated with reduced SG formation in response to combinatorial stress. This reduced SG sensitivity may contribute to therapy resistance and represents a potential vulnerability that could be therapeutically exploited.

### HSP70 downregulation enhances stress granule formation and restores Vemurafenib sensitivity in resistant melanoma cells

**Fig. 7:**
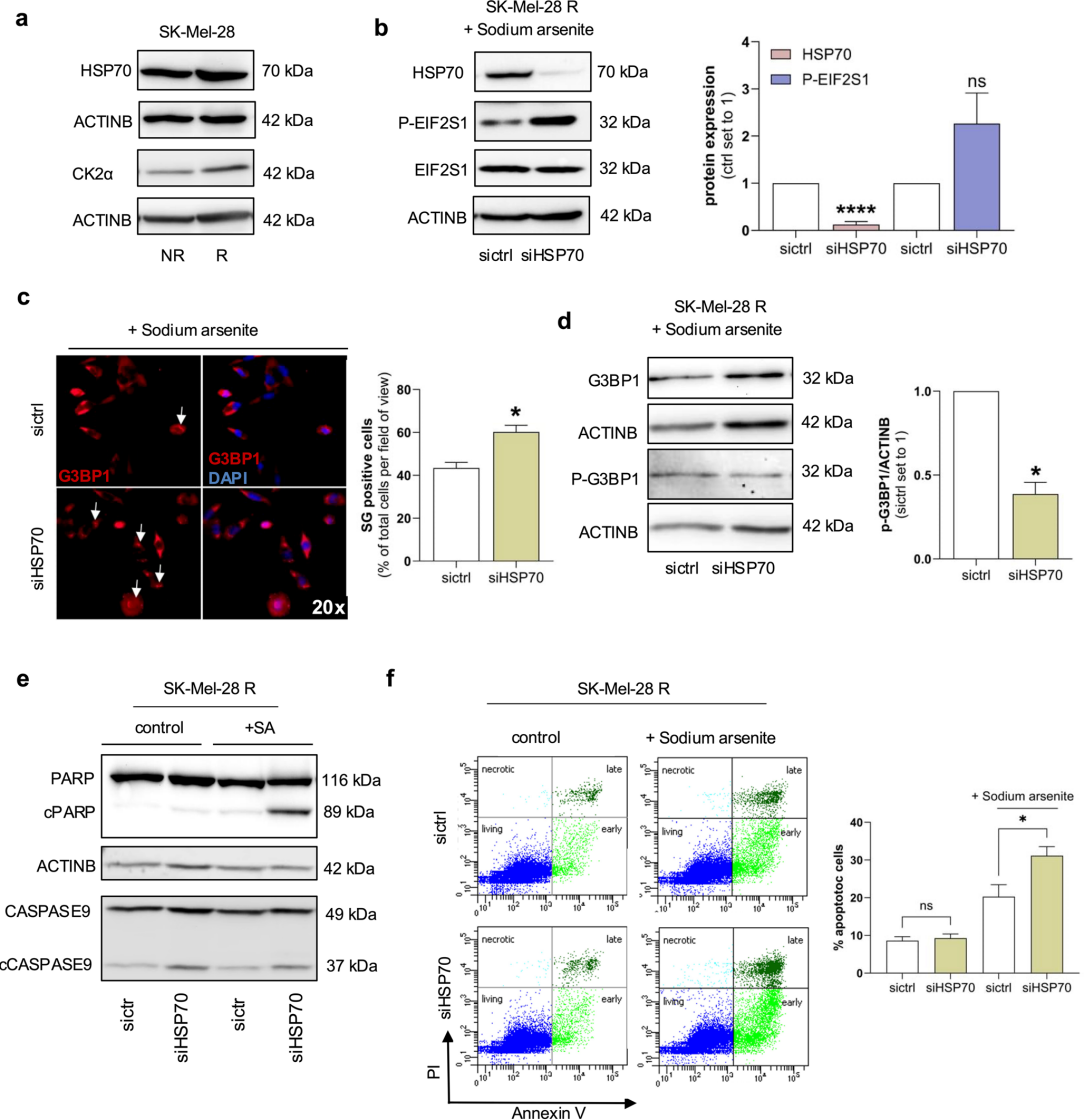
Role of HSP70 in vemurafenib-resistant melanoma cells. **(a)** Representative western blot analysis of HSP70 and CK2α protein expression in SK-Mel-28 non-resistant (NR) and vemurafenib-resistant (R) cells. ACTINB was used as a loading control. **(b)** Western blot analysis of HSP70 and phosphorylated EIF2S1 (p-EIF2S1) expression in SK-Mel-28-R cells transfected with HSP70 siRNA (siHSP70) or non-targeting control siRNA (sictrl) for 24 h. Total EIF2S1 and ACTINB were used as loading controls. Densitometric quantification from three independent experiments is shown. **(c)** Immunofluorescence staining of G3BP1 (red) in SK-Mel-28-R cells following sodium arsenite (SA) treatment (600 µM) for 24 h. Nuclei were counterstained with DAPI (blue). Quantification of SG-positive cells per field of view is shown as the mean of three independent experiments. **(d)** Western blot analysis of phosphorylated G3BP1 (p-G3BP1) in SK-Mel-28-R cells transfected with siHSP70 or sictrl, followed by SA treatment for 24 h. Total G3BP1 and ACTINB served as loading controls. Quantification from three independent experiments is included. **(e)** Western blot analysis of full-length and cleaved PARP and CASPASE9 in SK-Mel-28-R cells after transfection with siHSP70 or sictrl, with or without additional SA treatment. ACTINB was used as a loading control. **(f)** Flow cytometric analysis of apoptosis in SK-Mel-28-R cells after siHSP70 or sictrl transfection (24 h), followed by SA treatment. Data reflect one representative experiment of three independent replicates. Data are presented as mean ± SEM (*p < 0.05 and ****p < 0.0001; ns = not significant)

To further explore the role of HSP70 in stress granule (SG) dynamics and drug resistance, we examined its expression and functional relevance in Vemurafenib-resistant melanoma cells. Western blot analysis revealed a modest increase of HSP70 and CK2 protein levels in SK-Mel-28R cells compared to their non-resistant control cells (Fig. 7a). siRNA-mediated knockdown of HSP70 in SK-Mel-28R cells led to increased phosphorylation of EIF2S1, indicating an enhanced stress response (Fig. 7b). Immunofluorescence analysis also demonstrated that HSP70 depletion significantly elevated SG formation upon sodium arsenite (SA) treatment (Fig. 7c). Interestingly, this increase in SGs was accompanied by a reduction in phosphorylated G3BP1 (P-G3BP1), suggesting altered SG regulatory dynamics following HSP70 knockdown (Fig. 7d). We next assessed whether reduced HSP70 expression could modulate Vemurafenib sensitivity in resistant cells under stress conditions. Western blot analysis showed increased levels of cleaved PARP and cleaved caspase-9 (cPARP, cCASP9) following combined SA and Vemurafenib (SA/V) treatment in SK-Mel-28R cells, indicating enhanced apoptotic signaling (Fig. 7e). These findings were further confirmed by Annexin V/PI-based flow cytometry, which demonstrated a significant increase in apoptotic cells under combinatorial stress conditions (Fig. 7f). Collectively, our results suggest that downregulation of HSP70 and subsequent dephosphorylation of G3BP1 sensitize Vemurafenib-resistant melanoma cells to treatment, particularly under additional stress induced by SA. This highlights a potential therapeutic strategy to overcome resistance via modulation of SG-associated pathways.

## Discussion

The tumor microenvironment poses a significant barrier to cancer cell survival, characterized by hypoxia, nutrient scarcity, hyperosmolarity, and proteotoxic stress. These hostile conditions necessitate robust adaptive mechanisms to sustain malignant growth [14]. As tumor cells proliferate rapidly, they frequently outpace vascular development, resulting in hypoxic regions and elevated endoplasmic reticulum (ER) stress due to imbalances in protein folding and processing [28–30]. To cope, cancer cells reprogram their metabolism by enhancing glycolysis while attenuating mitochondrial oxidative phosphorylation, a shift that inadvertently leads to excessive production of reactive oxygen species (ROS) and a heightened dependence on antioxidant systems [31, 32]. Arsenical agents such as sodium arsenite (SA) exacerbate oxidative stress by inhibiting antioxidant enzymes, thereby increasing intracellular ROS and triggering the formation of stress granules (SGs), cytoplasmic aggregates that transiently sequester translational machinery and stress response proteins [33].

Notably, our in vitro and in vivo analyses revealed a near-complete absence of SGs in untreated melanoma cells, prompting deeper mechanistic exploration. Key to SG formation is the phosphorylation of eukaryotic initiation factor 2α (EIF2S1), a process that was robustly induced by SA in all melanoma cell lines tested, implicating activation of the HRI kinase pathway [13]. We found that SA strongly induced phosphorylation of eukaryotic initiation factor 2α (EIF2S1) in all tested melanoma cell lines, a hallmark of SG formation via the HRI kinase pathway. This finding contradicts earlier reports that suggested limited SG induction by SA in melanoma [34], establishing SA treatment as a reliable positive control in this context.

Despite elevated basal expression of G3BP1, a canonical SG nucleator, in both melanoma cells and normal human epidermal melanocytes (NHEMs), this alone was insufficient to drive SG assembly, highlighting tumor-type-specific regulation [14]. Neither hypoxia nor extracellular acidification (MES treatment) significantly induced SG formation in melanoma, diverging from patterns observed in breast and cervical cancers where such stresses are potent SG triggers [35, 36]. Furthermore, UVB-irradiated melanocytes failed to form SGs even following SA treatment, suggesting lineage-specific adaptations. This resistance may reflect the evolutionary pressure on melanocytes to manage chronic oxidative stress associated with UV exposure [37].

Recent studies have emphasized the role of G3BP1 serine 149 (S149) phosphorylation in modulating SG dynamics, where phosphorylation at this residue impairs G3BP1’s granule-nucleating capacity and impedes SG assembly, thereby affecting the cellular stress response in various cancer contexts [24, 38, 39]. Our results implicate constitutive phosphorylation of G3BP1 at S149 as a key mechanism of SG suppression in melanoma. We could show that Casein kinase 2α (CK2a) is responsible for modulating G3BP1 phosphorylation. Our data is in line with the study by Reineke et al., which could link CK2 activity to G3BP1 phosphorylation and thereby modulation of stress granules formation in osteosarcoma cells [25].

Notably, CK2α activity is aberrantly elevated in melanoma and has been linked to therapeutic resistance and tumor progression [27, 40, 41].

The negative regulatory effect of S149 phosphorylation on SG formation aligns with these findings [25].

In parallel, we observed that melanoma cells express high levels of the molecular chaperone HSP70 under basal conditions, a feature confirmed through analysis of The Human Protein Atlas and our experimental datasets. HSP70 is pivotal in maintaining proteostasis, disassembling protein aggregates in an ATP-dependent manner, and facilitating SG disassembly following cellular stress [18, 42]. YB-1, an established transcriptional activator of HSP70, is highly expressed in melanoma and may drive this constitutive expression [43–45]. Importantly, knockdown of HSP70 sensitized melanoma cells to SG formation and apoptosis, highlighting its functional role in stress resistance. Resistance to BRAF inhibition by vemurafenib was accompanied by diminished SG response to stress stimuli, but HSP70 silencing re-sensitized resistant cells, enhancing apoptotic responses. This underscores the therapeutic promise of targeting HSP70, as supported by previous findings showing synergy between HSP70 and MEK inhibitors in NRAS-mutant melanoma [46].

Collectively, our data show that melanoma cells employ at least two SG-suppressive mechanisms: constitutive G3BP1 phosphorylation and sustained HSP70 expression to maintain proteostasis and evade apoptosis. Disrupting these mechanisms may restore SG responsiveness and resensitize cells to therapy, particularly in drug-resistant settings. Notably, melanoma cells were sensitized to stress response inhibition only after sodium arsenate (SA) treatment, indicating that oxidative stress induction is required to expose this vulnerability. Although SA was used as a model compound, the findings support a broader therapeutic concept: combining oxidative stress–inducing agents with targeted inhibition of stress responses could enforce tumor cell dependency on adaptive pathways and create a therapeutic window, as also proposed by other studies [47–49]. This strategy, however, warrants further validation with additional agents and in vivo models.

## Supplementary Information

Below is the link to the electronic supplementary material.

Supplementary Material 1

Supplementary Material 2

Supplementary Material 4

Supplementary Material 4

## Data Availability

The datasets analysed during the current study are available in the GEO DataSets (GPL11154) repository, [https://www.ncbi.nlm.nih.gov/gds/?term=GSE284779].

## Abbreviations

SG: Stress Granule
SA: Sodium Arsenite
CK2: Casein Kinase 2 Alpha (CSNK2A1)
eIF2α: eukaryotic initiation factor 2 alpha
G3BP1: Ras GTPase-activating protein-binding protein 1
