# Supplementary material for: In long-lasting cellular stress phases of melanoma cells, stress granules are dissolved by HSP70
Source: Cell Mol Life Sci. 2025 Oct 28;82(1):366. doi: 10.1007/s00018-025-05939-8 (PMC12569246; doi:10.1007/s00018-025-05939-8)
Supplement: Supplementary file 1 — Supplementary Material 1 [file 18_2025_5939_MOESM1_ESM.pdf]

Primary Antibodies

Target	Dilutions Western Blot	Dilutions Immunofluorescence	Source of Supply
ACTINB	1:5000	1:1000	Themo Scientific
p-eIF2S1 (Ser51)	1:500	1:250	Abcam
eIF2S1	1:1000	1:500	Abcam
G3BP1	1:250	1:250	Sigma-Aldrich
p-G3BP1 (Ser149)	1:1000		Sigma-Aldrich
HSP70	1:1000		Dako
HIF1alpha	1:300		Novus Biological
CASPASE9	1:1000		Cell Signaling
PARP	1:1000		Cell Signaling
Cleaved PARP	1:1000		Cell Signaling
Cleaved CASPASE9	1:1000		Cell Signaling
CK2α	1:1000		Cell Signaling

Secondary Antibodies

mouse-HRP	1:1000	-	Themo Scientific
rabbit-HRP	1:1000	-	Themo Scientific
mouse-AP	1:3000	-	Themo Scientific
rabbit-AP	1:4000	-	Themo Scientific
Alexa Fluor 488 goat anti mouse	-	1:150	Themo Scientific
Alexa Fluor 488 goat anti rabbit	-	1:250	Themo Scientific
Alexa Fluor 594 goat anti rabbit	-	1:200	Themo Scientific
