# Supplementary material for: In long-lasting cellular stress phases of melanoma cells, stress granules are dissolved by HSP70
Source: Cell Mol Life Sci. 2025 Oct 28;82(1):366. doi: 10.1007/s00018-025-05939-8 (PMC12569246; doi:10.1007/s00018-025-05939-8)
Supplement: Supplementary file 2 — Supplementary Material 2 [file 18_2025_5939_MOESM2_ESM.pdf]

We used primary melanoma cell lines in different growth phases of radial growth (RGP) and vertical growth (VGP) to show examples of melanoma progression. RGP includes melanoma in situ. It has no mitoses, no metastatic potential , and a good prognosis. VGP is associated with greater potential for invasion and metastatic spread and a poor prognosis.

Cell Line	Resource Identification Initiative (Cellosaurus)	Source
Mel Juso	CVCL_1403	DSMZ, ACC 74
Mel Im	CVCL_3980	
Mel Ho	CVCL_1402	DSMZ, ACC 62
SK-Mel-3	CVCL_0550	DSMZ, ACC 321
SK-Mel-28	CVCL_0526	ATCC, HTB-72
Panc-1	CVCL_0480	ATCC, CRL-1469
Hep3B	CVCL_0326	ATCC, HB-8064
HepG2	CVCL_0027	ATCC, HB-8065
HT29	CVCL_A8EZ	ATCC, HTB-38
SW480	CVCL_0546	ATCC, CCL-228

Cultivated in Dulbecco's modified Eagle's medium (DMEM) supplemented with penicillin (400 units/ml), streptomycin (50 µg/ml) and 10 % fetal calf serum (all from Sigma-Aldrich, Steinheim, Germany). DSMZ, Leibniz Institute DSMZ-German Collection of Microorganisms and Cell Cultures; ATCC, American Type Culture Collection

Cell Line	Resource Identification Initiative (Cellosaurus)	Source
SBcl2	CVCL_D732	Meenhard Herlyn<br>The Wistar Institute, Philadelphia, USA
WM1158	CVCL_6785	
WM1366	CVCL_6789	
WM3211	CVCL_6797	
WM35	CVCL_0580	
WM793	CVCL_8787	
WM9	CVCL_6806	

https://wistar.org/research-discoveries/business-development/research-tools

The cell lines were cultivated in MCDB153 (Sigma-Aldrich) with 20% Leibovitz's L-15 (PAA Laboratories, Coelbe, Germany), 2 % FCS, 1.68 mM CaCl2 (Sigma-Aldrich), and 5 µg/ml insulin (Sigma-Aldrich).
